# Eco-evolutionary feedbacks generate bistability in population persistence under gradual environmental change

**DOI:** 10.64898/2026.08.05.743160

**Published:** 2026-08-08

**Authors:** Kuangyi Xu, Hao Shen

## Abstract

Understanding how populations persist in gradually deteriorating environments through evolution is a central question in ecology and evolutionary biology. Previous studies have primarily focused on identifying the critical rate of environmental change beyond which extinction is certain. However, the existence of a viable equilibrium when the rate is below the threshold does not guarantee that a population can survive the transient dynamics to reach it. Using a quantitative genetic model that explicitly incorporates feedback among population size, genetic variance, and mean trait evolution, we show that population persistence can exhibit bistability when the rate of environmental change is below the extinction threshold. Specifically, extinction still occurs if the initial population size and genetic variance fall below a critical level. The initial state also influences the eco-evolutionary dynamics, such that a temporary increase or decline in population size and/or genetic variance does not necessarily predict the ultimate fate of the population. Therefore, in addition to estimating the critical rate of environmental change for extinction, characterizing current population size, genetic variation, and the degree of maladaptation may improve predictions of extinction risk in deteriorating environments.

## Full Text Availability

The license terms selected by the author(s) for this preprint version do not permit archiving in PMC. The full text is available from the preprint server.

